# Increasing large scale windstorm damage in Western, Central and Northern European forests, 1951–2010

**DOI:** 10.1038/srep46397

**Published:** 2017-04-12

**Authors:** H. Gregow, A. Laaksonen, M. E. Alper

**Affiliations:** 1 Finnish Meteorological Institute, P.O. Box 503, Helsinki, FI−00101, Finland; 2 University of Eastern Finland, Department of Applied Physics, POB 1627, 70211 Kuopio, Finland.

## Abstract

Using reports of forest losses caused directly by large scale windstorms (or primary damage, PD) from the European forest institute database (comprising 276 PD reports from 1951–2010), total growing stock (TGS) statistics of European forests and the daily North Atlantic Oscillation (NAO) index, we identify a statistically significant change in storm intensity in Western, Central and Northern Europe (17 countries). Using the validated set of storms, we found that the year 1990 represents a change-point at which the average intensity of the most destructive storms indicated by PD/TGS > 0.08% increased by more than a factor of three. A likelihood ratio test provides strong evidence that the change-point represents a real shift in the statistical behaviour of the time series. All but one of the seven catastrophic storms (PD/TGS > 0.2%) occurred since 1990. Additionally, we detected a related decrease in September–November PD/TGS and an increase in December–February PD/TGS. Our analyses point to the possibility that the impact of climate change on the North Atlantic storms hitting Europe has started during the last two and half decades.

Studies of historical large scale storms have mostly focused on the meteorological parameters (e.g., wind, pressure, vorticity)^1^,^2^. In particular, investigations making use of meteorological reanalyses^3^,^4^,^5^ have found increases in the numbers and intensity of storms since the beginning of last century, as well as shifts in storm tracks, whereas studies utilizing *in situ* data have found no robust evidence for long-term increasing trend in storminess^6^,^7^,^8^,^9^,^10^,^11^ but rather a decrease until the mid 20^th^ century, and an increase after that. As pointed out by Feser *et al*.^2^, studies using non-meteorological proxies, such as forest damage, describing storm activity, can be particularly valuable, as they are independent of meteorological measurements and models, and can—therefore—be used to accept or reject hypotheses based on weather observations or reanalyses.

Forests and forested area are growing quickly in Europe^12^,^13^,^14^. Also forest damage has increased. Long-term forest loss climatologies have been published for Sweden (1901–2000)^15^ and Switzerland (1858–2007^16^ and 1891–2007^17^). Schelhaas *et al*.^18^ showed that there was an upward trend in storm damages to forests in 1850–2000 but concluded that “The two most probable reasons for the increase in storm damage have to do with changes in forest structure and increase in coniferous forests and growing stock which are partly a result of forest management”. With a somewhat different view from Schelaas *et al*.^18^, Cucchi & Bert^19^ stated that moderate PD caused by weaker storms can, to some extent, be influenced by the forest management related factors as described above but large scale damage can not. In line with Cucchi & Bert^19^, Usbeck *et al*.^17^, as well as Gardiner *et al*.^20^,^21^, have shown that for large forest damage to happen the storms need to cause gusty winds of around 40 ms^−1^ or more. On the other hand, Seidl *et al*.^22^ concluded, based on a multivariate statistical analysis, that forest change and climate change contributed more or less equally to the increased wind damage to forests in Europe.

In this paper, we present a systematic approach for combining forest growth^12^,^13^, storm-induced PD^21^, and storm count statistics from 1951 to 2010^22^ (http://www.efiatlantic.efi.int/portal/databases/forestorms/). All of the storms included in the analysis have caused PD/TGS of at least 0.012%, and they were validated to be of large scale (>500 km in diameter) with the reanalysed weather datasets provided by Wetterzentrale and NCAR/NCEP^23^. Climatic varibility and change are assessed analysing storm intensity as a function of NAO^24^ and as a function of time. We use information of PD from 17 countries in Western, Central and Northern Europe in 1951–2010. We also present statistical analyses of changes in the storm intensity time series between 1951 and 2010. Our main goal is to assess the storm intensity trends and their significance relative to the ongoing climate change.

## Results

### TGS and PD in Europe in 1951–2010

**Figure 1:**
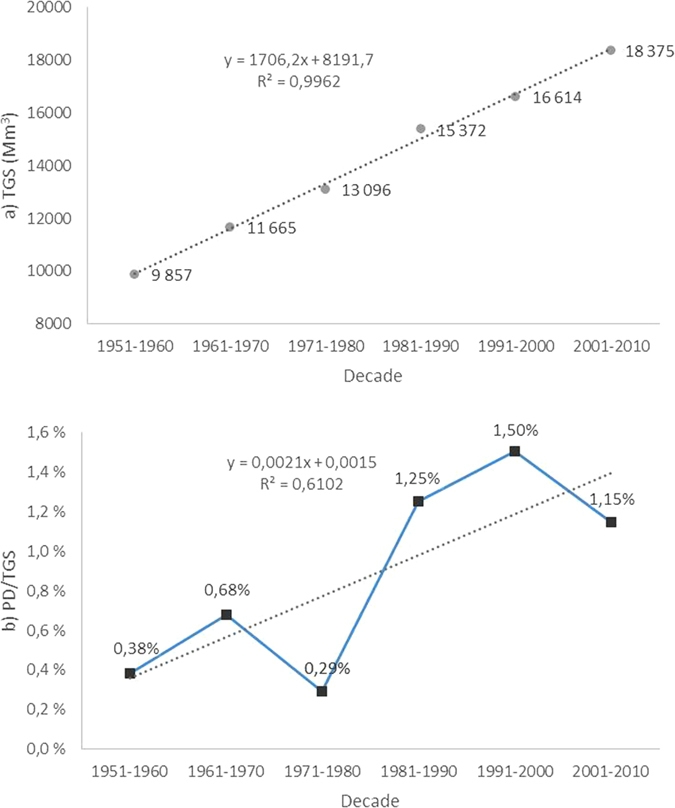
(**a**) Total growing stock (TGS) estimated based on Gold^12^ and FAO^13^ (**b**) and the decadal storm-induced PD in Western, Central and Northern Europe from 1951–2010, based on the FORESTORMS database^22^. Note that severe storm damages occurred in 1990, and had we defined the decades as 1950–59 etc. instead of 1951–1960 etc, the 4^th^ decade would have moved down below the trendline, and the 5^th^ decade would have correspondingly moved upward.

TGS has increased in Western, Central and Northern Europe from 1951 to 2010 (Fig. 1a) by nearly 100%. The dataset provided by Gardiner *et al*. (2010) totals around 960 Mm^3^ PD in 60 years. Forest losses have been greatest in Central Europe, where they have totaled approximately 340 Mm^3^ in 1951–2010. In Northern and Western Europe, the losses have been around 260 Mm^3^ and 290 Mm^3^, respectively. The most heavily affected countries have been France (≈260 Mm^3^), Germany (≈240 Mm^3^) and Sweden (≈220 Mm^3^). Largest individual damages have resulted from the storms Vivian and Wiebke (95 Mm^3^; 1990), Lothar and Martin (228 Mm^3^; 1999), Gudrun (77 Mm^3^; 2005), Kyrill (49 Mm^3^; 2007); and Klaus (44 Mm^3^; 2009). The bias caused by increased TGS values was removed from our analyses by using PD/TGS, rather than PD, as the proxy variable that indicates storm intensity. Additionally, in our analyses, we only focused on a validated set of large scale storms indicated by PD/TGS ≥ 0.012%. The decadal PD/TGS in Europe from 1951 to 2010 for the validated set of storms is shown in Fig. 1b. A comparison of the total European PD/TGS during the first decades (1951–1960) to the three most recent decades (1981–1990, 1991–2000 and 2001–2010, respectively) indicates that the damages have become 3–4 times as large as they were in the first decade of the period.

**Figure 2:**
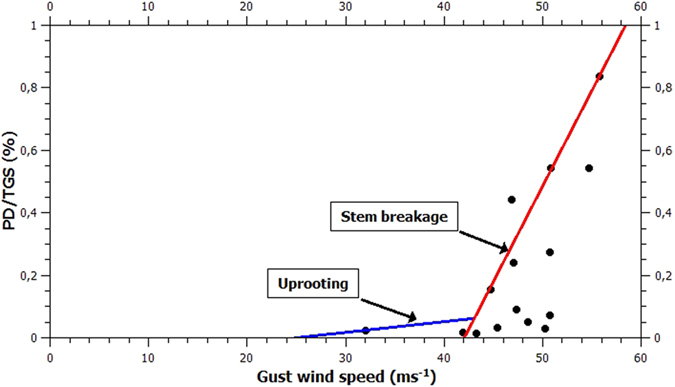
PD/TGS vs. gust wind speed for 15 storms. See text for details.

Usbeck *et al*.^17^ showed that forest losses caused by past storms in Swizerland are well correlated with maximum gust wind speeds (and poorly correlated with average wind speeds) measured during the storms. Spruce trees can be uprooted when gust wind speeds increase above 25 ms^−1^ (note, however, that uprooting may not occur if the soil is frozen, which is often the case in northern Fennoscandia in the winter). It has recently been shown^25^ that the critical wind speed at which trees break irrespectively of tree size or species is about 42 ms^−1^. Spruce trees that can be uprooted account roughly for 30–40% of the TGS in Europe. Thus, when the gust speeds exceed 42 ms^−1^, the potential for forest damages increases considerably. This can be seen from Fig. 2 showing PD/TGS vs. measured gust speeds for the 15 storms that can be found both in the FORESTORMS database^22^ and in the europeanwindstorms database^26^. The gust speeds are averages of ten highest wind speeds measured during the individual storms, read from plots of measured vs. reanalysed gusts shown at www.europeanwindstorms.org ((c) Copyright Met Office, University of Reading and University of Exeter. Licensed under Creative Commons CC BY 4.0 International Licence: http://creativecommons.org/licenses/by/4.0/deed.en_GB). The blue and red areas representing uprooting and tree breakage regimes, respectively, are meant to be illustrative rather than quantitative.

### NAO and storms

**Figure 3:**
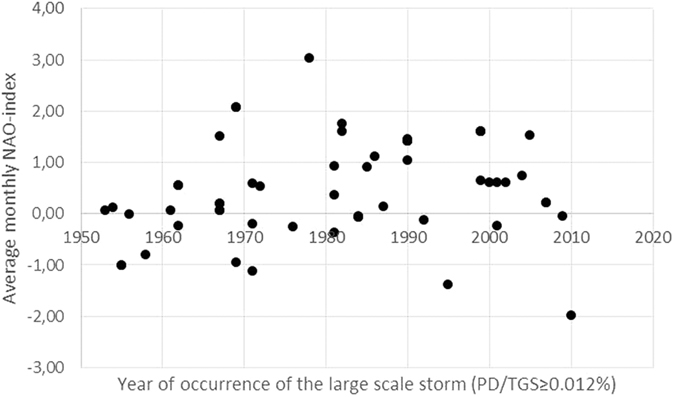
Large scale severe storms and concurrent monthly NAO-index.

Hanna *et al*.^7^, who studied long-term surface pressure records in Northwest Europe and the NorthernNorth Atlantic, and Allan *et al*.^27^, who studied storms over the British Isles, found that the correlation between NAO and storm activity is not constant over time. Our storm data collection (see SI for details) indicates that in the larger European scale, severe storm occurrence is biased towards positive NAO (see Fig. 3).

**Figure 4:**
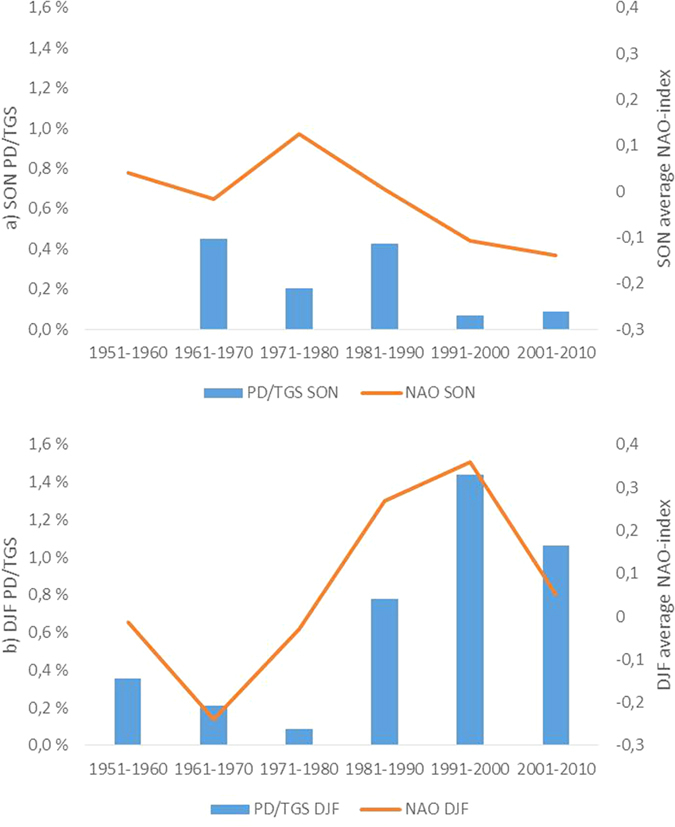
Decadal variation in PD/TGS and NAO in Europe in 1951–2010 during (**a**) autumn and (**b**) winter.

Figure 4a shows that the autumn (SON) PD/TGS has had a declining trend over the 60-year period (note, however, that there were almost no autumn PDs in the 1950s – although it remains a possibility that some of the FORESTORMS undated storms in fact occurred during SON). It is also apparent, from Fig. 4, that the correlation between autumn PD/TGS and the NAO index is much poorer than for winter (DJF) storms (the r^2^-value is, in fact, very close to zero). This is broadly in agreement with the findings of Allan *et al*.^27^, although they saw a weaker correlation between winter storms and NAO, and a slightly stronger correlation between autumn storms and NAO than we did. However, they looked at a limited area compared to us and, furthermore, defined the seasons differently from us (i.e., autumn as OND and winter as JFM).

It is clear from Fig. 4b that the PD/TGS of winter storms has increased during the six decades (p = 0.06) and that there is a correlation with the average NAO index. The r^2^ value of the correlation is 0.65; if the last decade is left out, the value is 0.75. While this could be a result of a sampling error in a data set of just six points, Hanna *et al*.^7^ and Allan *et al*.^25^ have also found that the correlation between NAO and storm activity is not constant over time.

### Storm intensity

**Figure 5:**
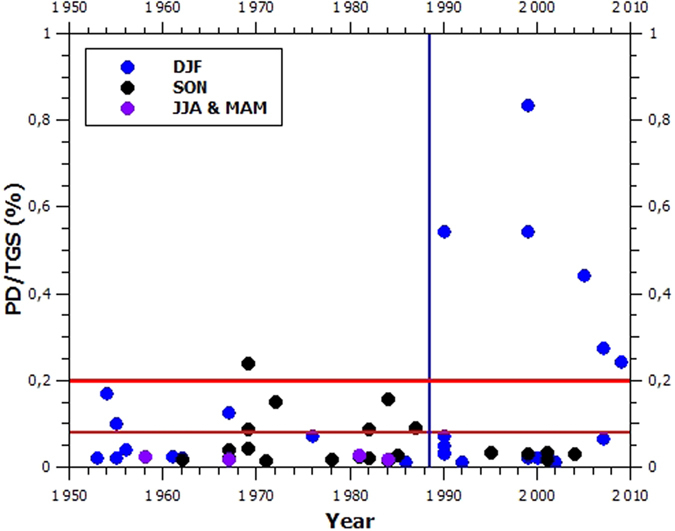
Time series of PD/TGS for all 56 storms considered in this work.

Figure 5 shows the complete time series of the 56 storms we consider in this work. We have divided the storms into three categories, separated by the red horizontal lines: destructive storms (PD/TGS < 0.08%), highly destructive storms (0.08% ≤ PD/TGS ≤ 0.2%), and catastrophic storms (PD/TGS > 0.2%). All but one of the seven catastrophic storms occurred since 1990. On the other hand, all eight highly destructive storms occurred before 1990. Furthermore, five out of the eight highly destructive storms and the only catastrophic storm prior to 1990 are SON, while all catastrophic storms after 1990 are DJF.

To answer the question whether the apparent change around 1990 is real or not, we utilize the generalized likelihood ratio test^28^ in determining whether PD/TGS time series contains a “change-point”, that is, a point after which the statistical behavior of the time series is significantly altered (note that we do not account for storm seasonality in this analysis). We assume that the PD/TGS values at each time step follow a Generalized Pareto Distribution^29^ (GPD), independent of each other. The null hypothesis states that the PD/TGS values all come from the same distribution, while the alternative states that there is a change-point at 1990. This test indicates a change-point with a p-value of 0.0001987. Furthermore, the most likely change-point (i.e. that which maximizes the likelihood ratio) is 1990. In terms of describing the change in intensity one could say that the highly destructive and catastrophic storms have intensified on average by a factor of 3.5 after 1990.

In addition to the aforementioned procedure, we also test the hypothesis of a change-point at 1990 in a model that explicitly takes into account the effects of NAO index on PD/TGS. The model in question has the form of a Generalized Linear Model (GLM), where the covariates are the yearly-averaged NAO indices and the dependent variables are the PD/TGS values. The shape parameter of GPD is modeled as 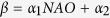 while the scale parameter as 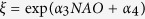 since it is a strictly positive quantity. A similar likelihood ratio test is performed to see if GLM coefficients change at 1990. Testing the whole dataset gives a significance of 0.01022 for a change-point at 1990. Separately considering the DJF months yield a significance of 0.04844, while no change is detected for SON. We stress that the p-values of these tests are not comparable, since the sample sizes differ vastly. This result implies that the NAO indicator itself does not explain the immense change occurring in PD/TGS values after 1990.

## Discussion

We made a systematic climatological study of 56 large scale windstorms based on data of significant forest damages (PD/TGS ≥ 0.012%) from 1951 to 2010 in 17 countries representing Western, Central and Northern Europe. The value of the study arises from the fact that the data is independent of meteorological observations – which, of course, do not provide complete coverage of the area – and of meteorological models.

Our results confirm that in the past three decades (1981–2010), PD/TGS in European forests caused by severe extratropical storms have become 3–4 times as large, per decade, as they were in the 1950’s, 1960’s and 1970’s. During the past 60 years, also the TGS has almost doubled in Europe. Unlike Schelhaas *et al*.^18^, we find that it is the increase in storminess itself that has caused much of the increased PD during the past decades, rather than changes in TGS alone. Our result is also in contrast to that of Seidl *et al*.^22^ who concluded that forest growth and forest management practices are affecting the increased PD as much as climate change in Europe. In particular, Seidl *et al*.^22^ considered that increased forest stand height and fraction of conifers has made European forests more vulnerable to windstorms. However, about 85% of all PD since 1990 have occurred as a result of individual catastrophic storms. In these storms, measured maximum gust wind speeds have been between 50–60 ms^−1^ (see www.europeanwindstorms.org)^26^. These facts, together with the step-wise nature of storm damage intensification in 1990, indicate that forest management practices have had at most a very minor influence on the increased PD.

Dawkins *et al*.^29^ found, based on insurance losses and the area of reanalysis-based windstorm footprints above 20 ms^−1^, that there has been a decline in European windstorms during the present century. At first sight, this appears contradictory to our finding of the change-point in 1990. However, one should note that Dawkins *et al*. only examined the period 1979 onward, and thus the decline is relative to the two last decades of the 20^th^ century that were dominated by the catastrophic storms of 1990 and 1999. Our data, on the other hand, suggests that the storminess in the first decade of this century was still at a clearly higher level than it was prior to 1990.

In accordance with earlier studies (e.g. Ulbirch and Christoph^30^, and references therein), we find that winter storms are correlated with the NAO index. We also find that autumn storms are not correlated with NAO index at all. Additionally, winter storm activity did not decrease as much in the 2000s, as could be inferred from the NAO index and as, for example, the storm index shown in Feser *et al*.^2^ would imply.

An interesting question relates to the reason for the decline in the autumn storms seen in Fig. 3. SON storms dominated over DJF storms in the 1960s and 1970s. The autumn PD/TGS was still high in the 1980s but declined during the past two decades of the period, while that of winter storms increased. Interestingly, the annual Arctic sea ice minimum also showed a decline in the 1990s and 2000s, simultaneously with the autumn storms. Francis and Vavrus^31^,^32^ suggested that Arctic amplification and sea-ice decline caused 500 hPa winds to decrease over the North Atlantic in October–December between 1980 and 2011. Moreover, a recent climate model study^33^ indicated reduced autumn cyclone activity in the Arctic in response to reduced sea ice. Thus, we feel that a possible connection between the autumn storminess and Arctic climate change is an issue that merits further study. It is also worth noting that in a study making use of historical ship logbooks as well as many land-based measurements and reports, it was foud that European storminess varied with warmth over the past few centuries, with damaging spring and autumn storms associated with colder periods^34^.

Concerning the reason behind the change-point in 1990, it is important to notice that there is a critical wind speed threshold at which severe forest damage begins. The change-point detected in the PD/TGS induced by windstorms could indicate that there is a change-point in the occurrence of critical wind speeds as well. Because the energy in the wind is proportional to the cube of the wind speed^35^, even a small change in the large scale windstorm intensity can have a substantial effect on the storm impacts. As NAO cannot explain the change-point, we suggest that climate change may play a role here, via its various impacts in the Arctic, and consequent changes in the large scale circulation and weather patterns^36^.

Although this work revealed new aspects of European storminess during the past six decades, more research is needed that combines oceanic, environmental and atmospheric variability and change to gain more certainty of the multiple impacts of climate change on storms.

## Methods

### TGS and PD

TGS statistics provided by Gold^12^ for the period of 1951–2000 and FAO^13^ for the period of 1991–2010 were used to form a longer time series of TGS in Europe (see SI for more details). To investigate the spatial changes in the occurrence and intensity of storms, we employed TGS raw data from 17 countries: Denmark, Finland, Norway, Sweden, Belgium, France, Ireland, Luxembourg, Netherlands, the United Kingdom, Germany, Czech Republic, Hungary, Poland, Slovakia, Austria and Switzerland. The storm-induced primary damage (PD) of the 17 countries of interest was obtained from the European Forest Research Insitute storm database (FORESTORMS^22^), which includes the dates of the storms, the storms’ descriptions, affected areas, PD reports and much more.

The researchers and experts who built the FORESTORMS database acknowledge that it may still lack storm damage data, and researchers are invited to add PD when or if more data are available. We notice that for instance, recent data from Finland is missing concerning the four consecutive summer storms in July-August 2010 causing 8 Mm^3^ PD to forests. However, the FORESTORMS database is currently the most comprehensive compilation of European storm-induced PD. Thus, it offers a unique source of data for assessing how the seasonal and decadal intensity and number of the most devastating storms have changed in Europe from 1951 to 2010 when using PD to forests as a key indicator of variability.

In our general assessment, only 0.2% of the damages found in the FORESTORMS database could not be used due to missing dates when assessing the general changes. In the storm specific analysis of the 56 storms, approximately 16% of the material was ruled out since the focus was on validated set of high impact large scale windstorms. The analyses were conducted on annual, seasonal and decadal scales. Trends and their significance were assessed using Microsoft Excel’s Regression Data Analysis Package Anova tests. Change-point analysis was performed using “changepoint” library version 2.2.2 in R statistical programming environment.

### Large scale storms

To validate the storms to be representative of large scale (>500 km in diameter), the dates of occurrence of primary damages and the reanalysed weather datasets provided by Wetterzentrale and NCAR/NCEP^23^ were used. The details of the individual storms are presented in Supplementary Table S4.

### Storm intensity and climate

Storm intensity was defined by PD/TGS/storm count. Storm intensity and storm count were investigated relative to the monthly NAO-index. Climatic variability of the severe large scale storms (PD/TGS ≥ 0.012%, storm diameter > 500 km) was assessed for seasons DJF and SON. The climatic assessment was carried out using annual seasonal averages but the results are presented on decadal scale to concentrate on climatic variability and change.

## Additional Information

**How to cite this article**: Gregow, H. *et al*. Increasing large scale windstorm damage in Western, Central and Northern European forests, 1951-2010. *Sci. Rep.* **7**, 46397; doi: 10.1038/srep46397 (2017).

**Publisher's note:** Springer Nature remains neutral with regard to jurisdictional claims in published maps and institutional affiliations.

## Supplementary Material

Supplementary Information
