# Supplementary material for: Increasing large scale windstorm damage in Western, Central and Northern European forests, 1951–2010
Source: Sci Rep. 2017 Apr 12;7:46397. doi: 10.1038/srep46397 (PMC5388877; doi:10.1038/srep46397)
Supplement: Supplementary Information [file srep46397-s1.pdf]

Supplementary Information

Increasing large scale windstorm damage in Western, Central and Northern European forests, 1951-2010

H. Gregow[1], A. Laaksonen[1,2], M.E. Alper[1]

[1] Finnish Meteorological Institute, P.O. Box 503, FI–00101 Helsinki, Finland
[2] University of Eastern Finland

Harmonizing the two datasets Gold and FAO and building decadal values for Northern, Western and Central Europe were accomplished by using linear regression and the data of Table S1. The overlapping period of 1990–2000 is shown in Fig.S1. There is very little difference in these two datasets.

Table S1. FAO-values of TGS (Mm^3) for the 17 countries in focus in years 1990, 2000, 2005 and 2010.

Nr	Country	1990	2000	2005	2010
1	Finland	1877,893	2082,112	2189,146	2189,146
2	Norway	701	809	898	987
3	Sweden	2791,2	3033,6	3234	3358
4	Denmark	64,9	74,3	106,3	108,4
5	Austria	947	1088	1159	1135
6	Belgium	128	157,4	164,1	167,9
7	Luxembourg	20,38	25,95	25,95	25,95
8	France	2077	2254	2512	2584
9	Ireland	61,5	69,6	70,6	74,3
10	Netherlands	52	61	65	70
11	United Kingdom	282	309	340	379
12	Germany	2815	3381	3458	3492
13	Czech Republic	625,2	698,8	735	769,3
14	Hungary	288,007	325,165	341,394	359,387
15	Poland	1485	1736	1909	2049
16	Slovakia	401,6	463,2	494,6	514,1
17	Switzerland	392	416	422	428

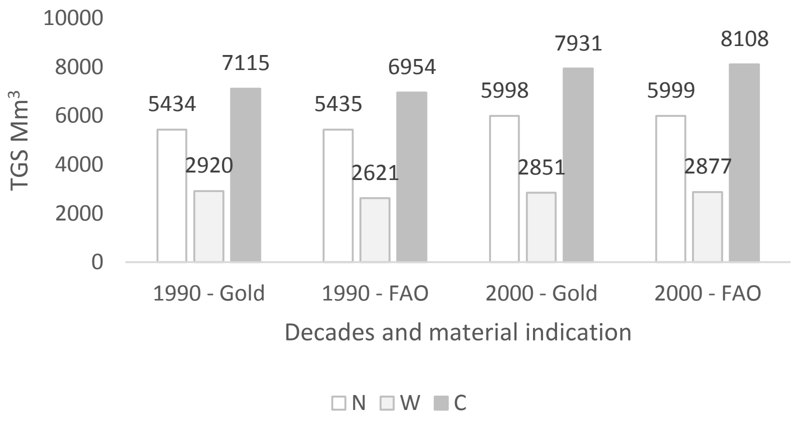

Fig.S1 Intercomparison of FAO and Gold reported TGS (Mm^3) values 1990-2010.

Estimated total growing stock of the countries for milestone years 1950, 1960, 1970, 1980, 1990, 2000 and 2010 in which Gold and FAO statistics have been combined for the last two decades. The decadal growth rate has been assessed based on the linear fit between the known milestone years.

Table S2. The regional average for Western, Central and Northern Europe is given as the cumulative sum in TGS.

Decade	1950	1960	1970	1980	1990	2000	2010
Sweden	1666,7	1905,57	2144,44	2383,31	2622,18	2861,05	3099,92
Finland	1314	1478,825	1643,65	1808,475	1973,3	2138,125	2302,95
Norway	329	414,365	499,73	585,095	670,46	755,825	841,19
Denmark	46,5	52,6765	58,853	65,0295	71,206	77,3825	83,559
Germany	741,87	1283,32	1824,77	2149,64	2799,38	3232,54	3492
Czech Rebublic	368	453	538	589	624	699	769,3
Hungary	155,31	189,16	223,01	243,32	283,94	311,02	359,387
Poland	1002,5	1170	1337,5	1438	1639	1773	2049
Slovakia	215,9	282,4	348,9	388,8	468,6	521,8	514,1
Austria	657,02	738,72	820,42	869,44	967,48	1032,84	1135
Switzerland	200	235	270	291	333	361	428
Belgium	54,3618	76,6281	92,5326	98,8944	127,5225	146,6079	168
France	1127,728	1384,924	1599,254	1892,986	2370,78	2172,976	2584
Ireland	3,88	12,64	19,94	22,86	36	44,76	74
Luxembourg	8,8	11,6	13,6	14,4	18	20,4	26
Netherlands	6,04	18,08	26,68	30,12	45,6	55,92	70
United Kingdom	85,872	150,216	203,836	225,284	321,8	410,2	379
**Cumulative TGS**	**7983,482**	**9857,125**	**11665,12**	**13095,65**	**15372,25**	**16614,45**	**18375,41**

Table S3. Based on the data in Table S2, the annual volume of TGS in Western, Central and Europe was estimated.

**Annual volume TGS (Mm^3)**		**1950**	**1960**	**1970**	**1980**	**1990**	**2000**
	**0**	**7983,482**	**9857,125**	**11665,12**	**13095,65**	**15372,25**	**16614,45**
	1	8170,846	10037,92	11808,17	13323,31	15496,47	16790,54
	2	8358,21	10218,72	11951,22	13550,97	15620,69	16966,64
	3	8545,575	10399,52	12094,28	13778,63	15744,91	17142,73
	4	8732,939	10580,32	12237,33	14006,29	15869,13	17318,83
	5	8920,303	10761,12	12380,38	14233,95	15993,35	17494,93
	6	9107,667	10941,92	12523,44	14461,61	16117,57	17671,02
	7	9295,032	11122,72	12666,49	14689,27	16241,79	17847,12
	8	9482,396	11303,52	12809,55	14916,93	16366,01	18023,21
	9	9669,76	11484,32	12952,6	15144,59	16490,23	18199,31

The large scale storms (PD/TGS ≥ 0.012%) could be identified in Western, Central and Northern Europe (Table S4). If the date was missing, the storm type could not be assessed, but the damage could be added to the cumulative value of a region. The greatest uncertainty remains in the storms from Sweden in 1954 and 1955. The overall damage report shows total damage of 18.45 Mm^3; however, it was clearly noted that the storm on January 3–4 in 1954 was the main reason for the damage. According to the SMHI (Swedish Meteorological and Hydrological Institute), the storm on January 3, 1954, caused 36 m/s storm winds and a storm on November 12, 1954, (with highest wind speeds of 33 m/s) was a strong one over the western coast. Primary damages of 18.45 Mm^3 in the EFIATLANTIC database are given for 1954 as a sum, with storm dates. Due to a lack of better knowledge, we divided the storm damage according to the following: 15 Mm^3 PD was marked for January 3–4, representing DJF in 1954, and PD of 3.45 Mm^3 was left for the period of SON in 1954 and DJF in 1955. Knowing the storms, wind speeds and reanalysed maps, it is very likely that the division is close to correct.

Table S4. The severe storms causing at least PD/TGS=0.012% damage were investigated based on year, region, primary damage, season, monthly mean NAO and for some storms also on gust wind speeds.

Gust wind ms^{-1}	TGS Mm^3	Year	Part of Europe	PD (Mm3)	NAO index	Season	PD/TGS
	8545,575	1953	West	1,8	0,06	DJF	0,000211
	8732,939	1954	Northern	15	0,11	DJF	0,001718
	8920,303	1955	Central	8,9	-1,01	DJF	0,000998
	8920,303	1955	Central	2	-1,013	DJF	0,000224
	9107,667	1956	Northern	3,7	-0,021	DJF	0,000406
	9482,396	1958	Central	2,38	-0,81	JJA	0,000251
	10037,92	1961	Central	2,5	0,06	DJF	0,000249
	10198,86	1962	Northern	2	0,55	DJF	0,000196
	10218,72	1962	Central	2,3	0,55	DJF	0,000225
	10218,72	1962	Central	2,1	-0,23	SON	0,000206
	11122,72	1967	Central	14	0,19	DJF	0,001259
	11122,72	1967	Central	2	0,19	DJF	0,00018
	11122,72	1967	Central	2	1,51	MAM	0,00018
	11122,72	1967	Northern	2,34	0,07	SON	0,00021
	11122,72	1967	Northern	4,47	0,07	SON	0,000402
	11484,32	1969	Northern	27,5	2,07	SON	0,002395
	11484,32	1969	Northern	5	2,07	SON	0,000435
	11484,32	1969	Northern	10	-0,96	SON	0,000871
	11808,17	1971	Northern	2	-1,13	DJF	0,000169
	11808,17	1971	Northern	2	0,58	SON	0,000169
	11808,17	1971	Northern	2	-0,2	SON	0,000169
	11951,22	1972	Central	18	0,54	SON	0,001506
	12523,44	1976	Central	9	-0,25	DJF	0,000719
	12809,55	1978	Northern	2,5	3,04	SON	0,000195
	13323,31	1981	Northern	3,9	0,92	DJF	0,000293
	13323,31	1981	Northern	3,9	0,36	MAM	0,000293
32	13323,31	1981	Northern	3,23	-0,38	SON	0,000242
	13550,97	1982	Northern	3	1,76	SON	0,000221
	13550,97	1982	Western	12	1,6	SON	0,000886
	14006,29	1984	Western	2,5	-0,07	JJA	0,000178
44,7	14006,29	1984	Western	2	-0,07	SON	0,000143
43	14006,29	1984	W, C	22	-0,06	SON	0,001571
	14233,95	1985	Northern	4	0,9	SON	0,000281
43,2	14461,61	1986	Central	2	1,11	DJF	0,000138
47,3	14689,27	1987	W,C,N	13,3	0,14	SON	0,000905
48,5	15372,25	1990	Western	8	1,04	DJF	0,00052
45,4	15372,25	1990	Central	5,1	1,41	DJF	0,000332

	15372,25	1990	Western	5,5	1,41	DJF	0,000358
50,7	15372,25	1990	Central	11,3	1,41	DJF	0,000735
50,8	15372,25	1990	Central	83,5	1,46	MAM	0,005432
	15620,69	1992	Northern	2	-0,13	DJF	0,000128
	15993,35	1995	Northern	5,8	-1,38	SON	0,000363
50,2	16490,23	1999	Northern	5	0,65	SON	0,000303
	16490,23	1999	Northern	3,5	1,61	DJF	0,000212
55,7	16490,23	1999	Western and Central	138	1,61	DJF	0,008369
54,7	16490,23	1999	Western	90	1,61	DJF	0,005458
	16614,45	2000	Northern	3,6	0,6	DJF	0,000217
	16790,54	2001	Northern	3	-0,24	SON	0,000179
	16790,54	2001	Northern	6,1	0,6	SON	0,000363
	16966,64	2002	Northern	2	0,6	DJF	0,000118
	17155,16	2004	Central	5,4	0,73	SON	0,000315
46,8	17318,83	2005	Northern	77	1,52	DJF	0,004446
	17847,12	2007	Northern	12	0,22	DJF	0,000672
50,7	17847,12	2007	Central	49	0,22	DJF	0,002746
47	18199,31	2009	Western	44,2	-0,06	DJF	0,002429
41,9	18375,41	2010	Central	3,59	-1,98	DJF	0,000195

42
